# Recalcitrant erythrodermic ichthyosis with atopic dermatitis successfully treated with Dupilumab in combination with Guselkumab

**DOI:** 10.1002/ski2.87

**Published:** 2021-12-22

**Authors:** M. Steinhoff, F. Al‐Marri, R. Al Chalabi, U. Gieler, J. Buddenkotte

**Affiliations:** ^1^ Department of Dermatology and Venereology Hamad Medical Corporation Doha Qatar; ^2^ Translational Research Institute, Academic Health System Hamad Medical Corporation Doha Qatar; ^3^ Weill Cornell Medicine‐Qatar Doha Qatar; ^4^ Qatar University, Medical School Doha Qatar; ^5^ Weill Cornell Medicine New York New York USA

## Abstract

**Background:**

Autosomal recessive congenital ichthyosis refers to a group of rare inherited disorders of keratinization and defective epidermal barrier resulting in varying clinical presentations and severities ranging from harlequin ichthyosis to congenital ichthyosiform erythroderma (CIE). Secondary atopic dermatitis (AD) can aggravate the disease state for CIE patients leading to recalcitrant CIE/AD with potentially unfavourable side effects and low tolerability.

**Aims:**

Here, we report about a 38‐year‐old male patient with severe CIE as well as AD over the last 30 years.

**Materials and Methods:**

The patients suffered from severe inflammation, pruritus and recurrent infections for decades without disease control and intolerable adverse events of previous therapies.

**Results:**

Dupilumab (targeting IL‐4Ra, 300 mg q2w) partially controlled pruritus, but only the combination of Dupilumab with Guselkumab (anti‐IL23p19) controlled both CIE and AD with markedly reduced inflammation, itch and recurrent infections. Guselkumab alone was not sufficient to treat the severe CIE/AD.

**Discussion:**

Further studies are required to assess the efficacy and safety of targeted therapies like Dupilumab/Guselkumab combination therapy in severe CIE/AD.

## Graphical abstract

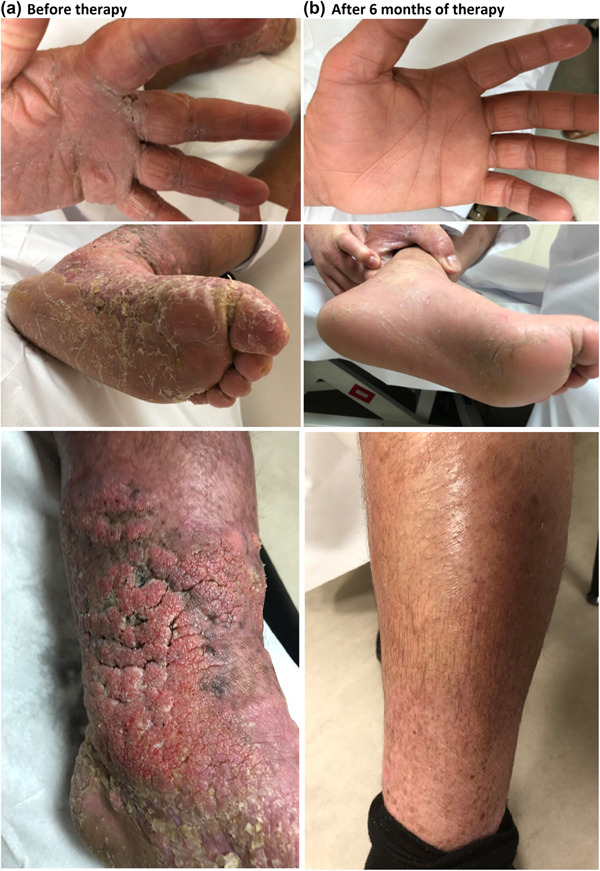

1

**What's already known about this topic?**

- Congenital ichthyosiform erythroderma is a severe inflammatory form of ichthyosis often associated with congenital ichthyosiform erythroderma (CIE)‐aggravating atopic dermatitis, which is renowned to be recalcitrant to therapy.

**What does this study add?**

- Combination therapy of Dupilumab and Guselkumab results in a rapid and sustainable improvement of itch, erythroderma, and eczema as well as all CIE symptoms. This observation suggests that IL‐4 receptor α and IL‐23p19 signalling are important traits in patients with combined severe CIE and severe atopic dermatitis.

## 2 INTRODUCTION

Congenital ichthyosiform erythroderma is an inherited autosomal‐recessive ichthyosis (ARCI) with xerosis presenting as fine white scales, erythrodermic episodes and marked palmoplantar keratosis.^1^ Additional skin manifestations of congenital ichthyosiform erythroderma (CIE) include erythroderma, blistering, infections and lymphoedema. In severely affected patients, eclabium, ectropion, scarring alopecia of the scalp and eyebrows can occur.^2^ So far, 13 gene mutations have been described in ARCI including *TGM1*, *NIPAL4, ALOXE3* and *ALOX12B*.^3^, ^4^, ^5^ Pathogenic variants of these genes are known or predicted to interfere with the normal skin structure resulting for instance in reduced stratum corneum ceramides and a disturbed skin barrier function, which precedes the formation of ichthyosis, xerosis and secondary atopic dermatitis (AD).^6^

Autosomal recessive congenital ichthyosis treatment is often challenging and mainly includes application of topical emollients, topical keratolytics, systemic retinoids and vitamin D derivatives.^6^ Unfortunately, therapies are often unsuccessful over years and promising therapeutic interventions such as enzyme replacement and cutaneous gene therapies not readily available as of yet.^6^ However, biological therapies, although successfully applied in the therapeutic regimen of AD, have so far not been tested in CIE.

We report a case of severe CIE and AD treated successfully after 30 years using Dupilumab and Guselkumab in combination demonstrating efficacy, safety and tolerability of a combined biological therapy that blocks two severe diseases and distinct immunological pathways.

## 3 REPORT OF CASE

**FIGURE 1:**
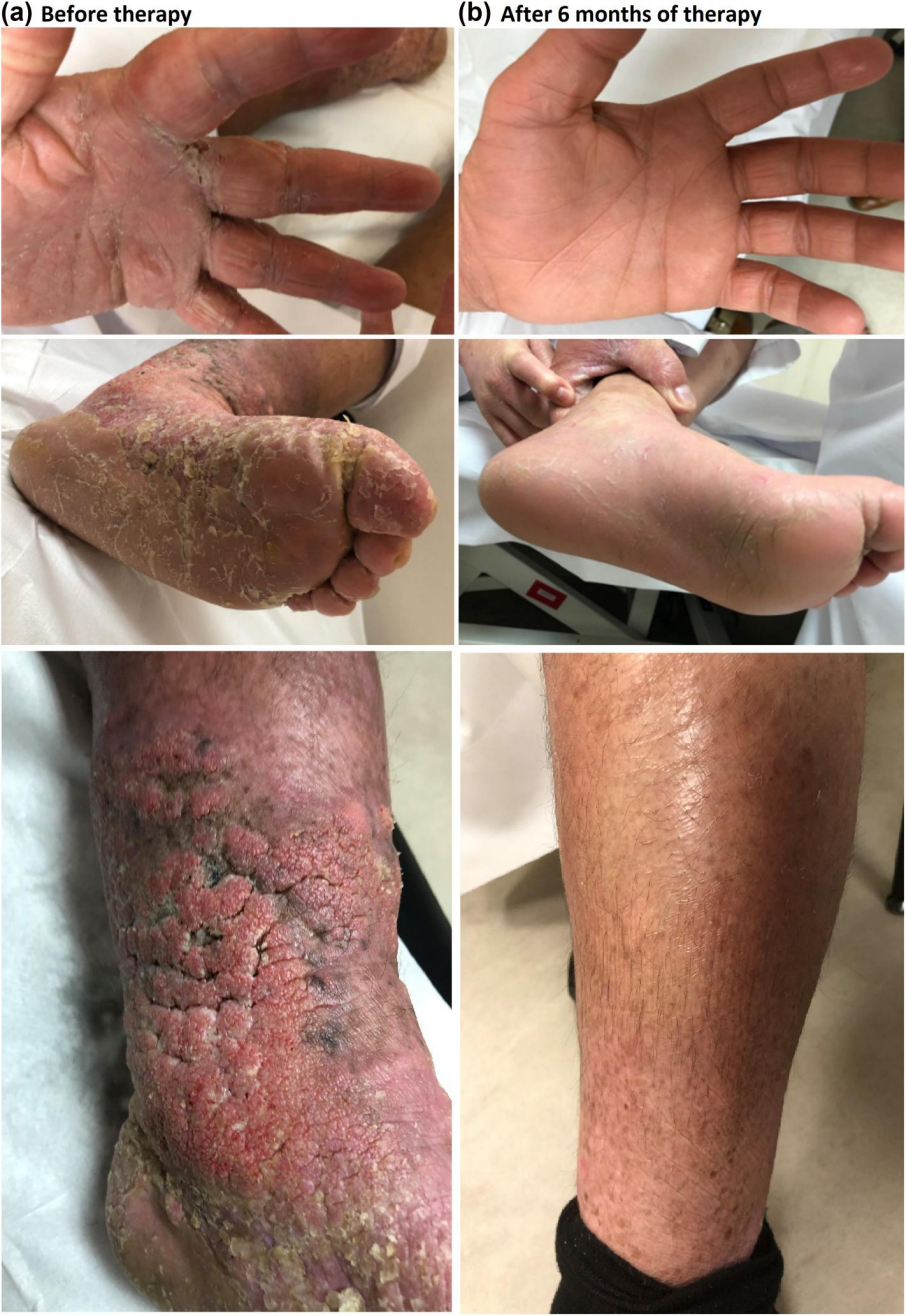
Clinical presentation of adult patient with congenital ichthyosiform erythroderma/atopic dermatitis before (a) and 10 weeks after Dupilumab/Guselkumab therapy (b) (for VAS score and SCORAD, see Table 1)

A 38‐year‐old Qatari male, with history of ichthyosis and atopic dermatitis since childhood, presented with severe itch, recurrent fungal and bacterial infections, lymphoedema and hyperkeratosis on extremities. Family history revealed ichthyosis and AD in four children and first‐degree relatives. Physical exam showed generalized erythema to erythroderma with severe scaly hyperkeratotic ichthyosiform plaques on lower extremities, hyperkeratotic palms and soles, and severe lymphoedema (Figure 1a). Laboratory findings before therapy showed elevated CRP (17 mg/L), monocytes (11%), and IgE (4362 UI/ml; *n* = 100 UI/ml). Eosinophils and neutrophils were within normal range (Table 1). Genetic testing revealed an R243H mutation in the CYP4F22 gene indicative of inherited erythrodermic ichthyosis. A skin biopsy revealed hyperkeratosis, profound spongiosis and acanthosis with marked dermal perivascular lympho‐monocytic infiltrate (not shown).

**TABLE 1:** Development of diagnostic marker under Guselkumab/Dupilumab combination therapy

	Naïve	Guselkumab/Dupilumab, 10 weeks	Guselkumab/Dupilumab, 80 weeks
VAS	10/10	1–2/10	1–2/10
SCORAD	81	6.6	8
BSA	90%	9%	10%
CRP	17 mg/L	12.1 mg/L	12 mg/L
IgE	4362 UI/ml	2203 UI/ml	1555 UI/ml
Monocytes	11%	8.5%	6.2%
Neutrophils	64.9%	54.1%	62%
Eosinophils	1.6%	1.6%	0.7%

Abbreviations: BSA, body surface area; CRP, C‐reactive protein; IgE, immunoglobulin E; VAS, visual analogue scale.

Multiple therapies failed to improve his severe condition over 30 years, among them topicals, cyclosporin A, methotrexate, and acitretin. Due to the AD aspect of the patient's CIE, the first line AD therapeutic Dupilumab 300 mg q2w was started with SCORAD (81), which improved his itch considerably, with mild improvement of his erythroderma, and eczema (SCORAD: 55) after 5 weeks. Because ichthyosis overlaps with psoriasis on transcriptome level,^8^ the psoriasis therapeutic Ixekizumab 80 mg q4W was added that rapidly improved erythroderma and eczema (SCORAD: 25), but aggravated fungal infection after 7 weeks. Therefore, we started Guselkumab 100 mg q8W, an alternative psoriasis therapeutic, together with dupilumab. Within 10 weeks, erythroderma, and eczema significantly improved his condition of AD as well as all symptoms of CIE without infections. After 6 months, described lesions had an IGA of 0/1 (SCORAD: 6.6) (Figure 1b). Both skin conditions reached an IGA0/1 over 80 weeks of monitoring.

## 4 DISCUSSION

Treatment of CIE, especially when associated with atopic dermatitis is still a challenge in dermatology. Biologic therapy is regularly used to treat atopic dermatitis,^7^ but rarely used in ichthyosis. Thus, a new innovative therapy to treat recalcitrant ichthyosis and AD is needed.

Because ichthyosis cytokine profile resembles that of psoriasis,^8^ targeting IL‐17 and IL‐23 is a rationale option to treat ichthyosis. Dupilumab alone was not sufficient to improve either eczematous lesions or ichthyosis in our patient, despite ameliorating itch. Ixekizumab (anti‐IL‐17A) improved patient's ichthyosis, however, worsened fungal infection. The switch to Guselkumab (anti‐IL23p19) resulted in a rapid, significant improvement of both ichthyosis and AD with good tolerability, together with dupilumab. Notably, this significant improvement was observed after 10 weeks of combined therapy and stable over 80 weeks without side effects.

Thus, fast improvement of the erythrodermic ichthyosis and eczema lesions using a combination of Dupilumab/Guselkumab indicate clinical evidence and proof‐of‐principle for an effective, safe, well‐tolerated treatment of CIE using anti‐IL‐23 and anti‐IL‐4 therapy in combination, if needed.

No reports so far exist about the use of Guselkumab in CIE, nor double therapy with two biologics for erythrodermic CIE and severe AD. Despite expensive, considering the costs of 30‐year failed treatment of disease and side effects, this treatment may be considered for the period of disease clearance. It cannot be excluded that Guselkumab alone or anti‐IL‐17 therapy may be also effective for milder ichthyosis. In this severe condition, addition of Guselkumab targeting IL‐23 and downstream IL‐17 dysregulation in CIE to Dupilumab for AD significantly improved both skin conditions without adverse events.^8^, ^9^

Here, for the first time to our knowledge, we report about the successful treatment of CIE/AD using two biologics targeting IL‐4Rα and IL‐23p19. Improvement was rapid, sustainable, and safe. The excellent safety profile and tolerability of the combined biologics therapy we report in this case needs examination in appropriate large‐scale clinical trials. However, combination therapy with these two biologics can be considered in severe cases of CIE/AD, and probably other severe dermatological disorders.

## CONFLICT OF INTEREST

Martin Steinhoff has served on advisory boards for Abbvie, Janssen, Sanofi/Regeneron, Pfizer, Eli Lilly, Galderma, Leo, MenloTx, and has been a consultant for Abbvie, Amgen, Novartis, Janssen, Pfizer, Eli Lilly, Sanofi, MenloTx, Union Tx, Galderma, Leo; and has received research funding from Galderma, Abbvie, Leo, Pfizer, Novartis. The other authors have no conflicts of interest to declare.

## AUTHOR CONTRIBUTIONS

**M. Steinhoff:** Conceptualization; Data curation; Formal analysis; Investigation; Methodology; Project administration; Resources; Software; Supervision; Validation; Visualization; Writing – original draft; Writing – review & editing. **F. Al‐Marri:** Data curation; Formal analysis; Investigation; Visualization; Writing – original draft; Writing – review & editing. **R. Al Chalabi:** Investigation; validation; Writing – review & editing. **U. Gieler:** Data curation; Formal analysis; Investigation; Methodology; Validation; Writing – review & editing. **J. Buddenkotte:** Conceptualization; Data curation; Investigation; Software; Writing – original draft; Writing – review & editing.

## Data Availability

The data points of this case report have been obtained at the Department of Dermatology and Venereology, Rumailah Hospital, HMC. Data available on request due to privacy/ethical restrictions.
